# Evaluation of Trace Metal Content by ICP-MS Using Closed Vessel Microwave Digestion in Fresh Water Fish

**DOI:** 10.1155/2014/201506

**Published:** 2014-03-13

**Authors:** Sreenivasa Rao Jarapala, Bhaskarachary Kandlakunta, Longvah Thingnganing

**Affiliations:** Food Chemistry Division, National Institute of Nutrition, Hyderabad 500007, India

## Abstract

The objective of the present study was to investigate trace metal levels of different varieties of fresh water fish using Inductively Coupled Plasma Mass Spectrophotometer after microwave digestion (MD-ICPMS). Fish samples were collected from the outlets of twin cities of Hyderabad and Secunderabad. The trace metal content in different varieties of analyzed fish were ranged from 0.24 to 1.68 mg/kg for Chromium in *Cyprinus carpio* and *Masto symbollon*, 0.20 to 7.52 mg/kg for Manganese in *Labeo rohita* and *Masto symbollon*, 0.006 to 0.07 mg/kg for Cobalt in *Rastrelliger kanagurta* and *Pampus argenteus*, 0.31 to 2.24 mg/kg for Copper in *Labeo rohita* and *Penaeus monodon*, 3.25 to 14.56 mg/kg for Zinc in *Cyprinus carpio* and *Macrobrachium rosenbergii*, and 0.01 to 2.05 mg/kg for Selenium in *Rastrelliger kanagurta* and *Pampus argenteus*, respectively. Proximate composition data for the different fishes were also tabulated. Since the available data for different trace elements for fish is scanty, here an effort is made to present a precise data for the same as estimated on ICP-MS. Results were in accordance with recommended daily intake allowance by WHO/FAO.

## 1. Introduction

Trace elements are found naturally at various levels in the hydrosphere, and many are required for physiological and metabolic processes of organisms [1]. These metals are also referred to as microminerals and are part of enzymes, hormones, and cells in the body. Insufficient intake of trace minerals can cause symptoms of nutritional deficiency. Due to industrialization, the number of factories and population has increased rapidly. The contamination of freshwaters with a wide range of pollutants has become a matter of concern over the last few decades [2–4]. It is unfortunate that we, human beings without realizing the consequences of pollution, do a lot of activities that terribly ruin the nature, resulting in the denial of healthy environment to our successors. Water contamination is one of the serious concerns that affect the marine ecosystem with high concentration of trace metals. According to [5] the coastal or river waters are contaminated by the dumping of industrial wastages. The metals accumulated in these waters pass through to human and infect the humans by direct consumption or through consuming the affected organisms like fishes. [6, 7] claim that when the level of trace metal concentrations exceeds the stipulated level it turns out to be toxic. Very recently, the work in [8] has stated that the higher level of metal concentration will bring shattering effect to the ecological balance by altering the range of organisms in water.

Trace elements are generally classified as either essential elements (Zn, Se, Cu, I, Mo, and Cr) or probably essential (Mn, Si, Ni, Bo, and Va). However both deficiency and excess intake of essential elements can be detrimental to human health. As top predators, fish may be the end reservoir of the bioaccumulation of trace elements in a food chain, causing them to be potentially hazardous to consume [9, 10]. But [11] has established the health benefits of advocated level of mineral consumption. They claim that deficiency of Zn will cause loss of appetite, growth retardation, skin changes, and immunological abnormalities. But [12] has stated that, though Zn has biological significance, excessive consumption of these kinds of metals will affect the humans. Apart from Zn, remaining trace metals also are playing an essential role in human daily life. The trace metal sewage from industries pollutes water and fishes in turn. The consumption of the affected fishes over a prolonged period will harm the health of humans.

Fortunately previous studies reveal that the trace metal concentration level in fishes is not that much alarming in South East Asian countries. The researchers have examined muscles, livers, and gills of fishes as these organs play different roles in bioaccumulation process [13]. But [14] says that the concentration level of metals in gills represents the level of metals in water, where they dwell. The concentrations of metals in liver represent their storage level. Metallothioneins (MTs) are the metal-binding proteins accumulated in livers, whereas [15, 16] assert that metal accumulation in the muscles of fishes is dangerous as they are the most edible part. They also established that environmental evaluation in aqua ecology shall be conducted in water, organisms, or sediments. Each of these components provides partial image of metal accumulation in the whole ecosystem.

In modern human nutrition, fish and fishery products have attracted considerable attention since they exhibit unique nutritional benefits for human health. It is well known that fish is one of the major sources of animal protein and has been widely accepted as a good source of other essential elements for the maintenance of a healthy body [17]. It is widely consumed in many parts of the world by humans because it has high protein content and low saturated fat and also contains omega fatty acids, which are well known to reduce cholesterol levels which in turn reduce the incidence of heart diseases, stroke, and preterm delivery [18–20]. The main functions of essential minerals include skeletal structure, maintenance of colloidal system, and regulation of acid-base balance. Minerals also constitute important components of hormones, enzymes, and enzyme activators [21].

The uptake of trace metals in fish occurs through food ingestion and water via the gills. Fish farming is the main activity in global aquaculture, a sector that has witnessed a steady increase in the past 15 years [22]. The food and agricultural organization (FAO) reported that of 154 million tons of fish that represented the worldwide production in 2009 [23] the fish production in India during 2010-2011 is estimated to be 8.29 million tons. Consumption of fish and other marine products has always been a major factor in the economy and nutrition of the population.

India is one of the leading countries in terms of fish, aquaculture, and marine food resources [24, 25]. Among the Asian countries India ranks second in aquaculture, fourth largest producer of fish, and one of the top leading exporters of seafoods. Several species of fresh water and marine water fish are available for human consumption. Information on micronutrient content in fresh water fish is very scarce [26].

Over the last few decades, there has been growing interest in determining trace metal and heavy metal contents in fresh water and marine environment and attention was drawn to the measurement of contamination levels in public food supply particularly in fish. Toxicological and environmental studies have prompted interest in the determination of trace metals and toxic elements in foods. Several reliable analytical methods were available for monitoring trace metals levels in fresh water and marine food samples, but Inductively Coupled Plasma Mass Spectrometer (ICP-MS), being the most sophisticated and reliable technique, was widely used for determination and quantification of trace metals in food samples. This study is focused on measuring the concentration level of selected metals (Mn, Zn, Cr, Co, Cu, and Se) in different species of fishes collected from twin cities of Hyderabad and Secunderabad. Moreover, proximate composition along with length and weight of the fish were also investigated to assess whether these fish were acceptable for human consumption.

## 2. Material and Methods

### 2.1. Reagents

All chemicals used were analytical grade quality. Ultrapure water was obtained from a Millipore water system (Millipore), ultrapure Nitric acid (HNO_3_, Merck) was used to digest the samples. Stock standard solutions of Chromium, Manganese, Cobalt, Copper, Zinc, and Selenium containing 10 *μ*g/mL in 2% HNO_3_ were procured from Sigma Aldrich, USA, and prepared as per the procedure [27]. The certified reference materials (CRM) were purchased from National Institute of Standard Technology (NIST-8436) and used for the standardization and validation of the method.

### 2.2. Collection of Fish Sample

Selected varieties of fresh water fish were collected from local markets of twin cities of Hyderabad and Secunderabad, Andhra Pradesh, India. The targeted species were commercial fish and shellfish available to consumers in Hyderabad. In the present study, fish and shellfish samples were collected by stratified random sampling procedure. This approach is the most suitable method for generating food composition database [28]. Collected fish samples were immediately curved in ice, kept in polystyrene boxes, and transported to laboratory to sustain freshness. Upon arrival to laboratory, fish and shellfish were individually measured for their total body weight and length.

### 2.3. Sample Preparation for Analysis

The fish samples were beheaded, gutted, washed, and filleted. The primary sample of each species was used to prepare a composite sample. Special care was taken to prevent metal contamination of the samples. All laboratory ware were soaked in 2 M HNO_3_ for 48 h and rinsed several times with distilled water and deionized water prior to use. Small size fish were pulverized with skin and the edible portion weight was recorded. Aliquots of edible tissue was taken for analysis of trace metals and proximate composition and stored in a freezer at −20°C till further analysis.

### 2.4. Microwave Digestion

The closed vessel microwave digestion system (CEM-MARS-USA) was used to digest homogenized fish samples (between 0.5 and 1.5 g) and placed in a Teflon digestion vessel with 3 mL of ultrapure HNO_3_ and 1 mL of hydrogen peroxide (H_2_O_2_-Merck). Sealed containers were placed in a microwave oven and heated according to the digestion program (program: power, 1600 W (100%); ramp time, 15 mins; temperature, 200°C; hold time, 15 mins; and cooling time, 15 mins). After digestion, sample solutions were cooled to room temperature then transferred quantitatively into acid cleaned 25 mL standard volumetric flasks and made up to 25 mL with double distilled deionized water and prepared under the same conditions as the calibration standards in 6% (v/v) HNO_3_. A blank digest was carried out in the same way [27].

### 2.5. Inductively Coupled Plasma Mass Spectroscopy (ICP-MS)

Chromium, Manganese, Cobalt, Copper, Zinc, and Selenium in fresh water fish were analyzed using ICP-MS (PerkinElmer Élan 9000-USA). For better operating conditions the ICP-MS was adjusted to nebulizer gas flow 0.91 L/min, radio frequency (RF) 1200 W, lens voltage 1.6 V, cool gas 13.0 L/min, and auxiliary gas 0.70 L/min [29].CRM samples were procured from National Institute of Standard Technology (NIST) and used for method standardization and validation. Standard graphs were drawn.Recovery study was also done.

## 3. Statistical Analysis

ANOVA was employed to test the difference between means of various fishes with regard to trace metals.

## 4. Result and Discussion

The weight and length of the available varieties of fresh water fish and shell fish were shown in Table 1. The fish varieties collected in the present study had shown variation in weights and lengths. Among the varieties studied,* Stromateus sinensis* with least length and weight (10–25 cm and weight is 0.05–0.15 kg) followed by* Penaeus monodon, Macrobrachium rosenbergii,* and* Rastrelliger kanagurta* with highest length and weight (15–35 cm and weight is 0.5–3.5 kg) followed by* Labeo rohita. *In the previous study conducted [30] the length of the fish and shell fish were gradually increased depending on the weight of the fish. Similar findings were observed in the fish collected for trace metal analysis in the present study.

### 4.1. Proximate Analysis

Proximate content of moisture, ash, and fat contents was determined using Association of Official Analytical Chemists [31]. Protein content (*N* × 6.25) was estimated using AOAC Kjeldahl method [32].

Proximate composition of fresh water fish was as shown in Table 2. Present study reveals that the moisture content of fresh water fish was diverse ranging from 75.89% in* Arius sona *and 81.11% in* Penaeus monodon*. Ash content was shown in the range of 0.81% to 1.68% in* Macrobrachium rosenbergii* and* Arius sona*. Total protein content was shown in ranges from 13.71% to 19.78% in* Stromateus sinensis* and* Pampus argenteus* and fat content ranged from 5.4% to 21.4% in* Labeo rohita* and* Arius sona,* respectively. These results were similar to those reported by [33] for sea bass, 76.72% moisture, 1.23% ash, 19.43% protein, and 4.81% fat. In another study the proximate composition values of fresh water fish range from 65 to 78% for moisture, 1.19 to 3.92% for ash, 14 to 18% for protein, and 1.53 to 5.41% for fat [34]. Reference [35] reported that the ranges of moisture in fish were from 63.52 to 70.71%, of ash from 1.35 to 1.66%, of protein content from 19.81 to 20.35%, and of fat levels from 6.10 to 15.11%, respectively, whereas in another study moisture content was 74.74%, ash content was1.53%, protein content was 18.80%, and fat content was 6.53% [36]. The proximate parameters also showed 69.91% for moisture, 1.22% for ash, 8.25% for protein, and 10.37% for fat [37]. The proximate composition values in fish were determined in the ranges of 72.22% of moisture, 1.57% of ash, 21.08% of protein, and 6.01% of fat [38]. It is known that the variations in the chemical composition of fish were closely related to nutrition, living area, fish size, catching season, and seasonal and sexual variations as well as other environmental conditions [39–41]. The variations in proximate parameters could be due to sexual and environmental conditions. The present author also published the proximate composition and heavy metal levels of Indian marine fish [42].

### 4.2. Trace Metal Contents in Fish

ICP-MS method was standardized and validated using certified reference material (CRM) purchased from National Institute of Standard Technology (NIST). Recoveries of trace metal contents in the present study were shown in Table 3 and the recovery ranges were shown from 95% to 106%. The summary of analyzed trace metal content in fresh water fish was presented in Table 4.

#### 4.2.1. Metal Bioaccumulation in Tissues

When fish are exposed to elevated metal levels in an aquatic environment, they can absorb the bioavailable metals directly from the environment via the gills and skin or through the ingestion of contaminated water and food. Metals in the fish are then transported by the bloodstream which brings it into contact with the various organs and tissues [43]. Fish can regulate metal concentrations to a certain extent after which bioaccumulation will take place [44]. Therefore, the ability of each tissue to either regulate or accumulate metals can be directly related to the total amount of metal accumulated in that specific tissue. Furthermore, physiological differences and the position of each tissue in the fish can also influence the bioaccumulation of a particular metal [45].

*(1)  Chromium. *Chromium (Cr) is a naturally occurring element found in rocks, animals, plants, and soil, predominantly in its insoluble trivalent form [Cr (III)]. Intense industrialization and other anthropogenic activities have led to the global occurrence of soluble Cr (VI), which is readily leached from soil to groundwater or surface water, in concentrations above permissible levels. The ecotoxicology of Cr (VI) is linked to its environmental persistence and ability to induce a variety of adverse effects in biologic systems, including fish. In aquatic ecosystems, Cr (VI) exposure poses a significant threat to aquatic life [46]. The Cr levels in the present study were shown in the range of 0.24–1.68 mg/kg in Cyprinus carpio and Masto symbollon. Cr levels in the literature have been reported in the range of 0.95–1.98 *μ*g/g [27]. In another study the range of Cr levels in fish was shown from 0.07 to 6.46 *μ*g/g dry weight in fish species from Iskenderun Bay [47]. Reference [48] states that the levels of Cr ranged from 0.97 to 1.70 *μ*g/g in canned fish. Cr content in Indian fish was shown in the ranges between 15 and 69 *μ*g/100 g [26]. However the Cr values in the present study agreed with FAO/WHO and the reported literature.

*(2)   Manganese.* Manganese is an essential element that can be found ubiquitously in the air, soil, and water, most of which is found in the liver, bones, and kidneys in human. This trace element is a cofactor for a number of important enzymes, including arginase, cholinesterase, phosphoglucomutase, pyruvate carboxylase, mitochondrial superoxide dismutase, and several phosphates, peptidases, and glycosyltransferases. In certain instances, Mn^2+^ may be replaced by Co^2+^ or Mg^2+^. Manganese functions with vitamin K in the formation of prothrombin. The levels of Mn in fresh water fish analyzed in the present study ranged from 0.20 to 7.52 mg/kg in* Labeo rohita* and* Masto symbollon*. Mn content in the literature has been reported in the range of 1.28–7.40 *μ*g/g in nine fish samples of Black and Aegean Seas, Turkey [27]. Reference [12] has reported that the Mn content was from 1.56 to 3.76 *μ*g/g in fish samples of the middle Black Sea in Turkey. Mn levels in Indian fish were shown in the ranges from 10 to 79 *μ*g/100 g [26].

*(3)  Cobalt.* Cobalt is beneficial for humans because it is a part of vitamin B12 [49]. However exposure to high levels of Co can result in lung and heart defects and dermatitis. Cobalt is used to treat anemia in pregnant women, because it stimulates the production of red blood cells. The levels of cobalt content in analyzed fish were shown in the range of 0.006–0.070 mg/kg in Rastrelliger Kanagurta and Pampus argenteus. Reference [50] has reported that the Co levels were in the range of 0.04–0.26 mg/kg. Cr levels in Indian fish purchased from internal markets were shown in the ranges of 0.02–0.67 mg/kg [51]. In the present study the cobalt levels were appropriate with the literature values. The Co content in different species of the fish shows wide fluctuations. The maximum Co content is observed in the species Pampus argenteus.

*(4)  Copper.* Copper in the blood exists in two forms: bound to ceruloplasmin (85–95%), and the rest “free,” loosely bound to albumin and small molecules. Free copper causes toxicity, as it generates reactive oxygen species such as superoxide, hydrogen peroxide, and the hydroxyl radical. These damage proteins, lipids, and DNA [52, 53]. Copper is an essential element in fish and is thought to be strictly regulated in the muscle tissue. In the present study the levels of copper in fresh water fish were found to be in the range of 0.31–2.24 mg/kg in* Labeo rohita* and* Penaeus monodon*. In the review of the literature Cu levels in fish samples have been reported in the range of 0.11–0.97 *μ*g/g in the northeast Atlantic and 0.04–5.43 *μ*g/g in Iskenderum Bay, northeast Mediterranean Sea, Turkey [47]. In Indian context Chandrashekar and Deosthale [26] have reported that the ranges of copper levels in fish were from 38 to 106 *μ*g/100g. Reference [54] has reported that the maximum copper level permitted for fishes is 20 mg/kg according to Turkish Food Codex. The reported value of copper in the present study was lower than the permissible levels.

*(5)  Zinc.* Zinc is an essential element in our diet. Zn is the most abundant element in the present study followed by Mn and Cu in the different species of the fish examined. It is generally believed that fish actively regulate Zn concentrations in their muscle tissue and as a result they do not reflect changes in ambient levels of this element in their environment [55]. Zn helps human body in maintaining sufficient immune system, cell division, wound healing, normal growth, and development during pregnancy and adolescence, to maintain stable state of the body, DNA synthesis, cellular metabolism, neurological function, reproduction, and so forth. Some hundreds of enzymes depend on this to catalyze chemical reactions. It plays a vital role in structure of proteins and cell membrane. Recently Zn has been found to play an important role in apoptosis which is nothing but a critical cellular regulatory process with implications for growth and development [56]. Zinc levels in the present study revealed that the values ranged from 3.25–14.56 mg/kg in* Cyprius corpio* and* Macrobrachium rosenbergii.* Zn content of different fishes in the literature has been reported in the range of 35 to 95 *μ*g/g [27]. Reference [57] has reported that the Zn ranges from 0.14 to 10.99 mg/kg in different varieties of fish. In another study Zn levels also have been reported in the range of 0.60 to 11.6 *μ*g/g in fish species from Iskenderum Bay [47]. Reference [26] has reported that the range of Zn levels in Indian marine fish was from 10 to 32 mg/kg. Zn content in the various fishes is lower than the reported Zn content of 21.7 mg/kg for the muscle tissue of* Lates calcarifer *from the coastal waters off Mumbai [58].

*(6)   Selenium.* Selenium also involves several biological functions in human body. It helps prevent oxidative stress by working together with a group of nutrients that prevent oxygen molecules from becoming too reactive. This group of nutrients includes vitamin E, vitamin C, glutathione, selenium, and vitamin B3. In many instances of heart disease, for example, where oxidative stress has been shown to be the source of blood vessel damage, low intake of selenium has been identified as a contributing factor to the disease. Similarly, in rheumatoid arthritis, where oxidative stress damages the area inside and around the joints, dietary deficiency of selenium has been shown to be a contributing cause [59]. In the present study the levels of Selenium in fresh water fish analyzed ranged from 0.10 to 2.05 mg/kg in* Rastrelliger kanagurta* and* Pampus argenteus*. Se content in the literature has been reported in the range of 0.36–0.46 mg/kg in fish species [60]. The levels of Se content in fresh water fish ranged from 0.24 to 0.28 mg/kg [35]. Reference [61] has reported that the Se levels were shown from 0.34 to 0.49 mg/kg in fish. The Se content in the present study was shown slightly higher than the reported value in literature.

Joint FAO/WHO Expert Committee on Food Additives recommends that for an average adult (60 kg body weight), the provisional tolerable daily intakes (PTDI) of copper is 3 to 4 mg and Zinc is 60 mg, respectively. The Zn level permitted for fish is 50 mg/kg, according to Turkish Food Codex. According to WHO the maximum tolerable levels of Selenium are 50 to 150 *μ*g/day, of Manganese 2–9 mg/day, of Chromium are 50 to 200 *μ*g and recommended daily intakes of Cobalt from 3 to 5 mg, respectively. As per the National Nutrition Monitoring Bureau (NNMB) an average household consumption of fish in Andhra Pradesh is 6 g/CU/day [62]. The consumption of fish is very low compared to the recommended levels of WHO/FAO [63].

## 5. Conclusion

In the present study the trace metal levels conform to FAO/WHO and the literature published values in fresh water fish except Selenium. However the estimated Se levels were higher in our samples than WHO/FAO recommended levels and those reported in the literature. The investigation of trace metals in fresh water fish demonstrated that Zn is the highest concentration in fish muscle followed by Cu, Mn, Se, Cr, and Co. It is known that a variation in the mineral composition of marine foods is closely related to seasonal and biological differences (species, size, dark/white muscle, age, sex, and sexual maturity), area of catch, processing method, food source, and environmental conditions. This study provides information on trace metal concentrations of fresh water fish consumed in twin cities of Hyderabad and Secunderabad and therefore provides an essential baseline data with which future levels may be compared and evaluated. Apart from other benefits, the data of the present study are also extremely useful to the scientific community and public officials involved in health risk assessment and management of environmental contaminants as well as a guide to the best course of action to restore ecosystems and, in turn, to preserve human health. However, it is just a selective fish investigation; metal contamination levels should be carefully monitored on a regular basis in more fish species, to detect the change in their accumulation patterns.

## Figures and Tables

**Table 1:** List of fresh water fish collected from markets with a narrow range of weight and length.

Local name	Common name	Scientific name	Edible portion/kg	Weight (Kg) (min.–max.)	Length (cm) (min.–max.)
Bommidayalu	Channa marulius	*Masto symbollon *	733	0.10–0.25	10–25
Ravva	Roho labeo	*Labeo rohita *	632	0.50–3.20	15–35
Chanduva	Pomfret white	*Stromateus sinensis *	630	0.30–1.50	13–35
Nallasandawah	Pomfret black	*Formio niger *	644	0.30–1.50	13–35
Jallalu	Cat fish	*Arius sona *	720	0.10–0.20	15–25
Chandamama	Silver pomfret	*Stromateus sinensis *	621	0.05–0.15	10–25
Bangaru teega	Gold fish	*Cyprinus carpio *	587	0.50–2.00	10–30
Korramenu	Soleole	*Rastrelliger kanagurta *	698	0.50–3.50	15–35
Royya	Tiger prawn	*Penaeus monodon *	570	0.10–0.30	05–15
Royya	Scampi prawn	*Macrobrachium rosenbergii *	408	0.10–0.20	05–15

**Table 2:** Proximate composition in fresh water fish—g%.

Local name	Scientific name	Moisture	Ash	Protein	Fat
Bommidalu	*Masto symbollon *	77.16 ± 1.2	0.997 ± 0.12	16.02 ± 0.52	12.05 ± 1.1
Ravva	*Labeo rohita *	78.29 ± 0.9	1.076 ± 0.11	17.49 ± 0.45	5.4 ± 0.85
Pomfret-White	*Pampus argenteus *	78.35 ± 1.5	1.067 ± 0.23	16.41 ± 0.78	13.45 ± 1.34
Pomfret-Black	*Pampus argenteus *	76.03 ± 0.82	0.956 ± 0.12	19.78 ± 0.85	6.8 ± 0.46
Jallalu	*Arius sona *	75.89 ± 1.21	1.685 ± 0.31	15.55 ± 0.23	21.4 ± 1.85
Chandamama	*Stromateus sinensis *	79.01 ± 1.43	0.978 ± 0.09	13.71 ± 0.13	15.55 ± 1.23
Gold fish	*Cyprinus carpio *	80.69 ± 1.89	0.976 ± 0.14	16.56 ± 0.53	5.48 ± 0.46
Korramenu	*Rastrelliger kanagurta *	78.42 ± 1.46	1.023 ± 0.21	18.92 ± 0.52	13.95 ± 1.78
Tiger prawn	*Penaeus monodon *	81.11 ± 0.82	0.817 ± 0.37	15.82 ± 0.37	10.00 ± 0.99
Scampi prawn	*Macrobrachium rosenbergii *	79.99 ± 0.79	0.816 ± 0.35	16.4 ± 0.42	10.8 ± 0.86

Values are mean ± SD, *n* = 6.

**Table 3:** Recovery study using certified reference material (CRM)—mg/kg.

Element	Analyzed value	NIST-certified value	% of recovery
Cobalt	0.008 ± 0.0006	0.008 ± 0.0004	100
Chromium	0.022 ± 0.008	0.023 ± 0.009	95
Copper	4.45 ± 0.56	4.30 ± 0.69	103
Manganese	14.92 ± 1.3	16 ± 1.0	99
Selenium	1.21 ± 0.1	1.23 ± 0.09	106
Zinc	22.42 ± 1.3	22.2 ± 1.7	100

Values are mean ± SD, *n* = 6.

NIST: National Institute of Standard Technology.

**Table 4:** Trace metal content in fresh water fish—mg/Kg.

Scientific name	Chromium	Manganese	Cobalt	Copper	Zinc	Selenium
*Masto symbollon *	1.68 ± 0.01	7.52 ± 0.02	0.03 ± 0.00	0.83 ± 0.01	12.2 ± 0.02	0.85 ± 0.02
*Labeo rohita *	0.27 ± 0.01	0.20 ± 0.03	0.01 ± 0.00	0.31 ± 0.00	6.96 ± 0.04	0.16 ± 0.01
*Pampus argenteus *	0.70 ± 0.04	1.84 ± 0.01	0.07 ± 0.00	0.62 ± 0.02	5.68 ± 0.01	0.56 ± 0.04
*Pampus argenteus *	0.98 ± 0.01	0.68 ± 0.00	0.02 ± 0.00	1.50 ± 0.07	7.57 ± 0.01	2.05 ± 0.01
*Arius sona *	0.81 ± 0.00	0.26 ± 0.00	0.02 ± 0.00	0.94 ± 0.01	5.13 ± 0.02	0.78 ± 0.00
*Stromateus sinensis *	0.87 ± 0.00	1.05 ± 0.02	0.008 ± 0.00	0.97 ± 0.03	5.18 ± 0.03	0.69 ± 0.01
*Cyprinus carpio *	0.24 ± 0.01	0.78 ± 0.00	0.03 ± 0.00	0.97 ± 0.05	3.25 ± 0.02	016 ± 0.00
*Rastrelliger kanagurta *	0.30 ± 0.00	0.23 ± 0.00	0.006 ± 0.00	0.50 ± 0.01	6.95 ± 0.03	0.10 ± 0.00
*Penaeus monodon *	0.67 ± 0.01	0.42 ± 0.00	0.01 ± 0.00	2.24 ± 0.01	11.44 ± 0.11	0.63 ± 0.00
*Macrobrachium rosenbergii *	0.92 ± 0.00	1.25 ± 0.03	0.02 ± 0.00	1.15 ± 0.01	14.56 ± 0.21	0.56 ± 0.00

Values are mean ± SD, *n* = 6.
